# Comparison of Cocoa Beans from China, Indonesia and Papua New Guinea

**DOI:** 10.3390/foods2020183

**Published:** 2013-05-21

**Authors:** Fenglin Gu, Lehe Tan, Huasong Wu, Yiming Fang, Fei Xu, Zhong Chu, Qinghuang Wang

**Affiliations:** 1 Spice and Beverage Research Institute, Chinese Academy of Tropical Agricultural Sciences (CATAS), Wanning, Hainan 571533, China; E-Mails: tlh3687@163.com (L.T.); gd_xiaogu@163.com (H.W.); liaoningdengta0000@yahoo.cn (Y.F.); xufei_0302054@163.com (F.X.); cz809@163.com (Z.C.); kjb3687@163.com (Q.W.); 2 National Center of Important Tropical Crops Engineering and Technology Research, Wanning, Hainan 571533, China; 3 Key Laboratory of Genetic Resources Utilization of Spice and Beverage Crops, Ministry of Agriculture, Wanning, Hainan 571533, China

**Keywords:** cocoa beans, average bean weight, cocoa butter content, total polyphenolic content, amino acids, e-nose

## Abstract

A survey on five kinds of cocoa beans from new cocoa planting countries was conducted to analyze each kind’s basic quality. The average bean weight and butter content of Hainan cocoa beans were the lowest, at less than 1.1 g, and 39.24% to 43.44%, respectively. Cocoa beans from Indonesia where shown to be about 8.0% and 9.0% higher in average bean weight and butter content, respectively, than that of Papua New Guinea and about 20.0% and 25.0% higher in average bean weight and butter content than Chinese dried beans, respectively. The average total polyphenolic content ranged from 81.22 mg/10 g to 301.01 mg/10 g. The Hainan 2011 sample had the highest total polyphenolic content, followed by the unfermented sample from Indonesia and the Papua New Guinea sample. The polyphenolic levels found in the Hainan 2010 sample were 123.61 mg/10 g and lower than the other three samples, but the Indonesian fermented sample had the lowest total polyphenolic content of 81.22 mg/10 g. The average total amino acid content ranged from 11.58 g/100 g to 18.17 g/100 g. The total amino acid content was the highest in the Indonesian unfermented sample, followed by the Hainan 2011 sample and the Papua New Guinea sample. The levels found in the Hainan 2010 sample were lower; the Indonesian fermented sample had the lowest total amino acid content.

## 1. Introduction

Cocoa is widely consumed in the form of chocolates, and the consumption rate is rising, because of the increasing popularity of chocolate confectioneries worldwide. Cocoa can also be found in beverages, cosmetics, pharmaceuticals and toiletries [1]. Cocoa beans begin as seeds in fruit pods of the tree *Theobroma cacao* L., where each fruit pod contains 30 to 40 beans embedded in a mucilaginous pulp. Raw cocoa has an astringent, unpleasant taste and flavor. To obtain the characteristic cocoa flavor and taste, raw cocoa must be fermented, dried and roasted [2]. The fresh cocoa bean is approximately composed of 32%–39% water, 30%–32% fat, 8%–10% protein, 5%–6% polyphenols, 4%–6% starch, 4%–6% pentosans, 2%–3% cellulose, 2%–3% sucrose, 1%–2% theobromine, 1% acids and 1% caffeine [3].

Commercial cocoa is obtained from beans that originated as seeds from the ripe pods of the plant, *Theobroma cacao*, which is cultivated in plantations in tropical regions throughout the world [4]. The three major cocoa growing regions are West Africa, Southeast Asia and South America. Indonesia produces about 15% of the world’s cocoa beans and ranks third in terms of international production. Indonesia has expanded its cocoa production and is now producing almost as much as Ghana. Indonesian cocoa production has the potential to substantially increase, but such increase would depend on local political and economic factors. As a member of the International Cocoa Organization (ICCO) and a signatory to the 2001 International Cocoa Agreement, Papua New Guinea is obliged to promote a sustainable cocoa economy. Cocoa trees may be grown in China, particularly in the southern region of Hainan province. The quality of the cocoa beans from that region was said to be similar to that of Indonesian cocoa beans [5].

The processing of cocoa beans comprises two major steps, namely, fermentation and drying. At cocoa plantations, fermentation and drying may primarily be looked upon as a curing process to stabilize the fresh beans through the microbial degradation of the firmly adhering, perishable pulp and through drying [6].

Genotype, soil, climate and harvest conditions, as well as processes, such as fermentation, drying and roasting, have important effects on the characteristics of cocoa. Unfermented and partly fermented cocoa beans are the beans of the *Theobroma cacao* L., which are dried without previously being fermented or partly fermented or by using improper procedures. Research has shown that these beans do not develop any chocolate flavor when roasted and are excessively astringent and bitter [7,8]. Malaysia is currently making use of these types of beans, which are imported from Indonesia, especially for cocoa liquor, powder and cocoa butter production. Cocoa manufacturers usually blend the unfermented and partly fermented beans with fully fermented beans to obtain the desired flavor characteristics and to reduce the excessive astringency and bitterness.

So far, comprehensive studies have yet to be conducted on the total content variation of phenol and flavonoid and of gallic acid and epicatechin. Moreover, no study has explored the content and composition of free amino acids in commercially fermented and dried cocoa from different origins. To address this gap in the literature, the present study is conducted by showcasing the variations in fermented, dried cocoa samples from different countries. This paper also reports on the content and composition of total and free amino acids of cocoa samples originating from three countries. The aim of the present study is to assess the influence of unfermented, fermented and dried cocoa beans from box fermentations performed by different countries in the last two years.

## 2. Experimental Section

### 2.1. Materials

Gallic acid, epicatechin and Folin-Ciocalteu phenol reagent were obtained from Sigma Chemical Co., Ltd. (St. Louis, MO, USA). Methanol and acetonitrile (HPLC grade) were purchased from Sigma-Aldrich Trading Co., Ltd. (Shanghai, China). All other chemicals used were of analytical grade and were obtained from Sinopharm Co., Ltd. (Shanghai, China).

*Theobroma cacao* beans of different geographic origins (Papua New Guinea, Indonesia and China) and of the Trinitario (hybrids of Criollo and Forastero) type were used in this study and were supplied by the Spice and Beverage Research Institute (Hainan, China) and Huadong Cocoa Co. Ltd. (Wuxi, China), and the cacao beans from Papua New Guinea and Indonesia were gotten from the main producing region and were the varieties cultivated popularly there. Fermented cocoa beans from Papua New Guinea were named C1, and fermented and unfermented cocoa beans from Indonesia were named C2 and C3, respectively. Fully ripe Hainan cocoa pods (*Theobroma cacao* L.) were handily harvested in 2010 and 2011 and were fermented in a box for six days. The fermentation mass was turned manually every day by transferring the coca pods from one box to another. Cocoa beans that underwent six-day fermentation were collected and sundried until a 7% moisture content was reached. The beans (named C4 and C5) were then stored in a refrigerator for later use. The five sundried samples, in turn, were roasted in an oven at 160 °C for 30 min in the laboratory and were milled using a Retsch blender (Restch, Haan, Germany) to obtain cocoa liquor. The five samples were marked as CR1, CR2, CR3, CR4 and CR5, respectively. 

The frozen cocoa bean samples were lyophilized (Labconco, Kansas, USA) until dry or until the beans became brittle and easily broken. The lyophilized beans were deshelled, degermed and ground using a Retsch blender (Restch, Haan, Germany). Small pieces of solid carbon dioxide were added occasionally to prevent cocoa lipids from melting, due to frictional heat caused by grinding.

### 2.2. Measurement of Average Bean Weight and Fat

The cocoa samples were placed in a ventilated oven at 60 °C until the constant dry weight of the bean components (beans and nibs) was achieved. The dry weights of the samples were then recorded. The results were shown as the gross weight and net weight of one cocoa bean. 

The lyophilized dry cotyledons were crushed, and 5.0 g portions were extracted repeatedly in a Soxhlet apparatus (Soxtec 2050, Foss, Hoganas, Sweden) with 500 mL of petroleum ether (b.p. 40–70 °C). Fat content was determined and expressed as a percentage of weight [9].

### 2.3. Measurement of Color and Absorbance at 420 nm

A 5.0 g portion of ground cotyledons was boiled in 45 mL of water and homogenized at 5000 rpm for 45 s. The homogenate was filtered and then centrifuged (1500× *g*, 15 min). The supernatant was determined at 420 nm using a UV-Vis Shimadzu UV-1601 spectrophotometer (Tokyo, Japan) [10]. 

Color was measured using an X-rite colorimeter (Xrite Inc., Grand Rapids, MI, USA). The results were expressed in the L*a*b* colorimetric system, according to the International Commission of Illumination, in which a color can be defined conventionally by three numerical parameters: sample luminance L* (quantity of reflected light), chromatic coordinated a* (red-green axis) and b* (yellow-blue axis) [11]. 

### 2.4. Measurement of Total Phenolic Content and Total Flavonoid Content

The defatted residue (cocoa powder) was air dried and stored at −20 °C before being extracted in boiling water for 1 h at a concentration of 20 mg/mL^−1^. After cooling at room temperature, the samples were centrifuged, yielding the final extract for analysis. 

The total phenolic phytochemical concentration was measured using the Folin-Ciocalteu method reported by [12]. Afterwards, 1 mL of appropriately diluted samples and a standard solution of gallic acid were added to a 25 mL volumetric flask containing 9 mL of ddH2O. A reagent blank using ddH2O was prepared. One milliliter of Folin-Ciocalteu phenol reagent was added to the mixture and was then shaken. After 5 min, 10 mL of 7% Na_2_CO_3_ solution was added prior to mixing. The solution was then immediately diluted to a volume of 25 mL with ddH2O and then mixed thoroughly. After incubation for 90 min at 23 °C, the absorbance relative to that of a prepared blank at 750 nm was measured using a spectrophotometer (Shimadzu UV-1601, SHIMADZU, Tokyo, Japan). The total phenolic contents of the samples were expressed in milligrams per serving of gallic acid equivalents (GAE). All samples were prepared in five replications. 

The total flavonoid concentration was measured using a colorimetric assay developed by [13]. One milliliter of appropriately diluted sample was added to a 10 mL volumetric flask containing 4 mL of ddH2O. At time zero, 0.3 mL of 5% NaNO_2_ was added to each volumetric flask; at 5 min, 0.3 mL of 10% AlCl_3_ was added; and at 6 min, 2 mL of 1 M NaOH was added. Each reaction flask was then immediately diluted with 2.4 mL of ddH2O and then mixed. The absorbances of the mixtures upon the development of pink color were determined at 510 nm relative to a prepared blank. The total flavonoid contents of the samples were expressed in milligrams per serving of epicatechin equivalents (ECE). All samples were prepared in five replications.

### 2.5. Measurement of Gallic Acid (GA) and Epicatechin (EC) Content

The (−)-epicatechin and gallic acid content were determined and quantified using the modified method of [14]. Dried cocoa samples were ground in a blender. Pieces of dry ice were added to the beans to prevent the cocoa lipids from melting, due to frictional heat caused by grinding. After grinding, the powder was sieved through a 710 μm screen. Powdered samples were defatted for 16 to 18 h using petroleum ether (b.p. 40–70 °C) as solvent. The samples were dried in a vacuum oven at 65 °C for 5 min and then stored in the dark inside a desiccator over silica gel prior to the extraction procedures. 

Concentrations of epicatechin and gallic acid were determined through High Performance Liquid Chromatography (HPLC, Agilent 1260 Infinity, Agilent, Waldbronn, Germany) and ultraviolet (UV, Agilent 1260 Infinity, Agilent, Waldbronn, Germany) detection (280 nm). Before analysis, defatted cocoa (4 g/L) was dissolved in 90% (v/v) water with 2% (v/v) acetic acid (pH 2.5) and 10% (v/v) acetonitrile, placed in an ultrasonic bath for 10 min and then filtered using a 0.45 μm cellulose filter. The mobile phase, at a flow rate of 1.0 mL/min, consisted of water plus acetic acid (pH 2.5, eluent A) and acetonitrile (eluent B) with the following gradients: 0.0 min, 90% A and 10% B; 20.0 min, 85% A and 15% B. Quantification was performed through external calibration with standard solutions of epicatechin and gallic acid. Results were expressed in milligram components per 10 g of cocoa product.

### 2.6. Measurement of Total Amino Acids

The defatted residue or cocoa powder was hydrolyzed with 6 M hydrochloric acid at 110 °C for 24 h under vacuum. The hydrolysate was submitted to an automated online derivatization with *O*-phthalaldehyde and reversed phase high performance liquid chromatography (RP-HPLC) analysis in an Agilent 1100 (Agilent Technology, Palo Alto, CA, USA) assembly system using a Zorbax 80A C18 column (4.6 i.d. × 180 mm) running at 0.5 mL/min. The results acquired were analyzed with the aid of ChemStation for LC 3D software (Agilent Technology, Palo Alto, CA, USA).

### 2.7. Measurement of Free Amino Acids

Determination of free amino acids was carried out using the extraction method of [15], with slight modifications. Only l-amino acids were quantified as cocoa flavor precursors. Seven hundred milligrams of defatted powder and 1.4 g of polyvinylpyrrolidone (PVP) were homogenized for 5 min at 0 °C in 15 mL distilled water and then adjusted to pH 2.5 using glacial acetic acid. The mixture was then centrifuged at 13,000× *g* for 15 min and then filtered through Whatman No. 4 filter paper. The filtrate was made to reach 50 mL using distilled water. Twelve milliliters of acetone and dl-alpha-amino-*n*-butyric acid (AABA, internal standard) were added to 3 mL of the filtrate. The mixture was then mixed thoroughly using a Polytron homogenizer, kept at room temperature for 30 min and centrifuged at 13,000× *g* for 15 min. Acetone was then removed by streaming with nitrogen gas. The amino acids were converted into phenylthiocarbamyl (PTC) amino acids using phenyl isothiocyanate (PITC). Twenty microliters of sample extract were used. The free amino acids were separated using RP-HPLC with gradient elution at a flow rate of 0.8 mL/min. Free amino acids were detected at 254 nm. Solvent A of the gradient elution was acetate buffer at pH 5.7, and solvent B was acetonitrile:deionized water (60:40). The gradient elutions were as follows: 0 min, 100% A, 0% B; 5 min, 75% A, 25% B; 13 min, 52% A, 48% B; 13.5 min, 0% A, 100% B; 16.5 min, 0% A, 100% B; 17 min, 100% A, 0% B; and 22 min, 100% A, 0% B. A Waters Pico-Tag Free Amino Acids Column (3.9 mm × 300 mm i.d., Waters, Millipore Corporation, Milford, MA, USA) was used for the analysis, which was employed at a temperature of 37 °C.

### 2.8. Measurement of Flavor with Electronic Nose (E-Nose)

The e-nose (model Gemine, Alpha M.O.S., Toulouse, France) with MOS chambers equipped with six sensors (LY/AA, LY/gCT, T30/1, P30/2, T70/2, PA/2) was connected to an auto sampler (CTC 100). A change in mass of a chemical compound caused a change in electrical resistance, as indicated by each sensor.

A 3.0 g mashed sample was placed into a glass vial. Using a crimping tool, each sample vial was sealed with a fitted cap and septum. Each vial was sealed tightly to prevent leaks and uncharacteristic decrease in sensor signal. The sample vials were then placed in the instrumental tray for further analysis. In this study, the sample was incubated at 50 °C for 10 min. The data collected using the e-nose were analyzed using principal component analysis (PCA) to differentiate the cocoa beans.

### 2.9. Statistical Analysis

One-way single-factor analysis of variance (ANOVA) was performed using SPSS software (version 16.0, SPSS Inc., Chicago, IL, USA). The *F* ratio was used to determine statistical significance at *p* < 0.05. A multiple-comparison test using Fisher’s least significance difference (LSD) was conducted. PCA was employed to describe the variability of sensory data.

## 3. Results and Discussion

### 3.1. Average Bean Weight and Butter Content

The gross weight and net weight of Indonesian cocoa beans were found to be higher than that of the beans from Papua New Guinea and China, which was the lightest (Table 1). Genotype, soil, climate, field management and harvest conditions have important effects on the characteristics of cocoa [7]. Papua New Guinea and China only began to plant cocoa in recent years; hence, the planting conditions for cocoa plants are not laid out effectively. The percentage of nib in Chinese cocoa beans was the highest among the samples (Table 1). The difference was caused by the thinner shell of Chinese cocoa beans compared with cocoa beans from Papua New Guinea and Indonesia.

**Table 1:** The average weight and butter content of a cocoa bean.

Samples	Gross weight/g	Net weight/g	Butter content (%)
C1	1.2124 ^a,b,c^ ± 0.0572	1.0757 ^a,b,c^ ± 0.0346	45.86 ^a,c,d^ ± 5.42
C2	1.3000 ^a,b,c^ ± 0.0143	1.1115 ^a,b,c^ ± 0.0261	53.67 ^a^ ± 4.81
C3	1.3946 ^b^ ± 0.0049	1.1713 ^b^ ± 0.3148	49.85 ^a,b,d^ ± 3.18
C4	1.1020 ^a,c^ ± 0.0997	1.0980 ^a,c^ ± 0.1582	39.24 ^c,d^ ± 4.25
C5	1.0225 ^c^ ± 0.0741	1.0062 ^c^ ± 0.2653	43.44 ^b,c,d^ ± 5.13

Mean values assigned with a common letter within the same column are not significantly different according to Duncan’s multiple range test at the 5% level.

The butter content of the dried beans of unfermented and fermented cocoa beans is shown in Table 1. Butter content varied from 39.24% to 53.67% for both fresh and fermented cocoa beans. Among the five dried samples studied, Indonesian cocoa beans showed higher butter content than both Chinese and Papua New Guinea dried beans. Zak and Keeney [16] reported that on average, fat, the main storage component of cocoa beans, comprises 53% to 58% of the cotyledon dry weight. These values are typical of fermented beans, but in the present study, the values varied among samples, genetic and geographic origins and climate. Demonstrably, ambient temperature and stress caused by heat or drought affect biosynthesis. In turn, the final composition of triacylglycerols and the melting and crystallization characteristics of cocoa butter are affected [17].

### 3.2. Color and Absorbance at 420 nm

After measuring the tristimulus color of cocoa beans obtained from unroasted and roasted cocoa beans (Table 2), L* did not change progressively after the roast, but fermentation did reduce the L* of cocoa beans obtained from C2 and C3. Both a* and b*, however, increased progressively after the roast, and higher incremental rates were observed in unfermented and fermented Indonesian cocoa beans after roasting. Great differences in a* and b* values were also observed in the cocoa beans of Papua New Guinea and Indonesia compared with those exhibited by Chinese fermented beans. This finding agrees with the spectral measurement (absorbance at 420 nm), wherein the spectral changes were higher in unfermented cocoa beans than in the fermented beans. The differences in the polyphenol oxidase activity and the phenolic compounds in unfermented and fermented cocoa beans were suspected to influence color formation intensity. If the fresh beans were dried without any fermentation, the nib would be slaty and grey in color, rather than brown or purple-brown, which is the color of fermented dried cocoa beans [18]. 

**Table 2:** The absorbance at 420 nm and the tristimulus L*, a* and b* values of cocoa beans.

Samples	A 420 nm	L^*^	a^*^	b^*^
C1	0.053 ± 0.021	42.22 ^a,b^ ± 0.243	5.240 ^a,b,c^ ± 1.165	3.327 ^a^ ± 1.183
C2	0.058 ± 0.038	45.93 ± 1.043	6.793 ^c^ ± 0.902	7.420 ^d^ ± 1.121
C3	0.887 ^a^ ± 0.022	47.42 ^c^ ± 0.904	4.940 ^a,b^ ± 0.242	6.783 ^c,d^ ± 0.193
C4	0.161 ^b^ ± 0.005	41.30 ^a^ ± 0.667	3.787 ^a^ ± 0.191	3.327 ^a^ ± 0.424
C5	0.195 ^c^ ± 0.005	41.52 ^a^ ± 1.187	4.633 ^a^ ± 1.166	2.323 ^a^ ± 1.020
CR1	0.217 ^d^ ± 0.002	43.59 ^b^ ± 0.621	6.393 ^c,b^ ± 0.263	5.367 ^b,c^ ± 0.194
CR2	0.218 ^d^ ± 0.004	43.67 ^b^ ± 0.501	7.457 ± 0.410	6.943 ^c,d^ ± 0.611
CR3	0.087 ^e^ ± 0.003	49.31 ^d^ ± 1.165	6.807 ^c^ ± 0.564	9.357 ± 1.310
CR4	0.048 ± 0.001	41.17 ^a^ ± 0.943	4.830 ^a,b^ ± 0.327	4.153 ^a,b^ ± 0.468
CR5	0.144 ^b^ ± 0.004	40.59 ^a^ ± 0.861	5.363 ^a,b,c^ ± 0.631	3.835 ^a,b^ ± 0.190

Mean values assigned with a common letter within the same column are not significantly different according to Duncan’s multiple range tests at the 5% level.

During fermentation of cocoa beans, polyphenols diffuse with cell liquids from their storage cells and undergo oxidation and complexation into high molecular mass, mostly insoluble, tannins. Anthocyanins are rapidly hydrolyzed into anthocyanidins and sugars (galactose and arabinose) by glycosidases. This process accounts for the bleaching of the purple color of the cotyledons. Polyphenol oxidases convert the polyphenols (mainly epicatechin and free anthocyanidins) into quinones. Polyphenols and quinones form complexes with other polyphenols, proteins and peptides. This process decreases the solubility and astringency of cocoa beans and gives rise to the brown color typical of well-fermented cocoa beans. The trismiclus L*, a* and b* values are reduced during roasting, because of Maillard reactions [18].

### 3.3. Total Phenolic Content and Total Flavonoid Content

**Figure 1:**
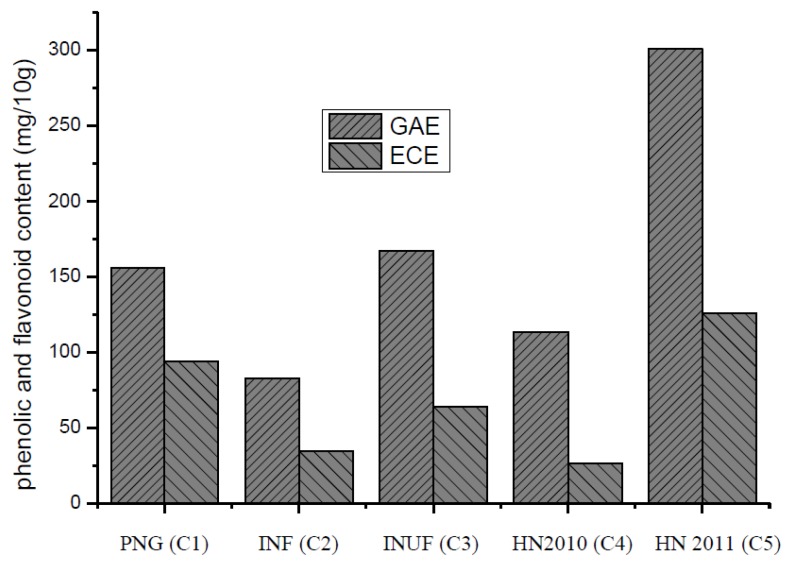
Total phenolic and flavonoid content of a cocoa bean, expressed as milligrams of gallic acid equivalents (GAE) and epicatechin equivalents (ECE) per 10.0 g, respectively.

The levels of total polyphenolic content were determined using the Folin-Ciocalteu method and were expressed as gallic acid equivalents (Figure 1). The average total polyphenolic content ranged from 81.22 mg/10 g to 301.01 mg/10 g. The total polyphenolic content was the highest in the Hainan 2011 sample, followed by the Indonesian unfermented sample and the Papua New Guinea sample. The levels found in the Hainan 2010 sample were higher than those found in the Indonesian fermented sample, which had the lowest total polyphenolic content. During fermentation, polyphenol concentrations of beans decrease, because of diffusion through water release and through further oxidation and condensation of the polyphenol compounds [19]. In the study, the polyphenol content of the dried beans further decreased after drying. Although season will not influence polyphenol contents of freshly harvested cocoa beans, weather conditions (microclimate and sunlight) and light intensity (position of pods on the tree, direct sunlight) seem to be important [20]. In general, the presence of polyphenols in cocoa beans is dependent on several factors, including the degree of pod ripeness, cocoa variety, processing and storage [21].

The levels of total flavonoid content were determined through the colorimetric assay method and were expressed as epicatechin equivalent (Figure 1). The average total flavonoid content ranged from 35.03 mg/10 g to 126.21 mg/10 g. The total flavonoid content was the highest in the Hainan 2011 sample, followed by the samples from Papua New Guinea and Indonesia, unfermented. The levels found in the Indonesian fermented sample were higher than those found in the Hainan 2010 sample, which had the lowest total flavonoid content. Recently, cocoa bean polyphenols have attracted a lot of attention, because of their potential benefits on human health. The health-promoting effects of polyphenols are believed to be the result of the relatively high antioxidant activity of these compounds, which protect people from chronic diseases by reducing oxidative damage. Cocoa polyphenols have been reported to have a wide range of biological properties, including modulating eicosanoid synthesis, increasing nitric oxide synthesis, lowering the rate of low-density lipoprotein oxidation, inhibiting platelet activation, stimulating the production of anti-inflammatory cytokines and inhibiting the production of certain pro-inflammatory cytokines [22].

### 3.4. Gallic Acid (GC) and Epicatechin (EA)

**Figure 2:**
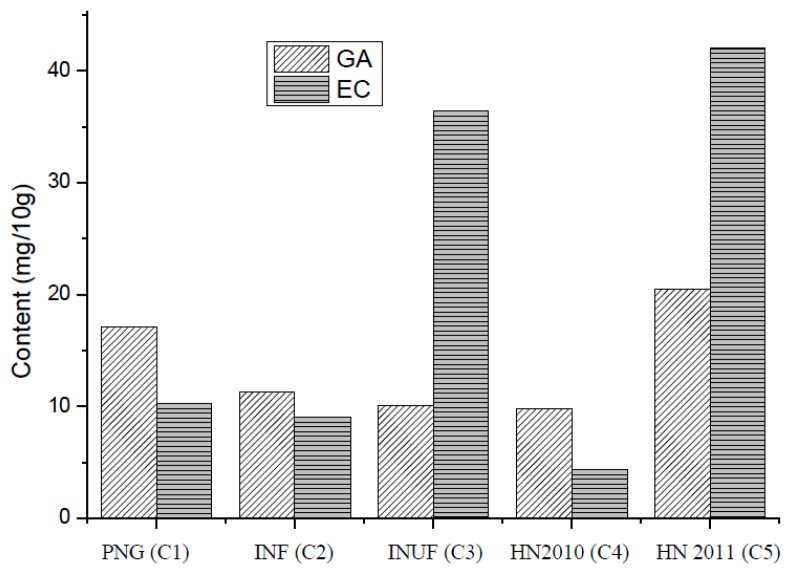
The content of gallic acid and epicatechin per 10.0 g of cocoa.

The predominant polyphenols identified in the freeze-dried defatted cocoa bean were catechin and epicatechin. Epicatechin represented 2% to 4% of the dry mass of defatted powder, whereas catechin was about 0.05% to 0.1% of the defatted bean. Epicatechin is the main polyphenol found in cocoa beans [20]. The gallic acid and epicatechin contents of the cocoa bean samples are shown in Figure 2. Hainan 2011 cocoa beans had the highest epicatechin content, followed by the Indonesian unfermented cocoa beans for water extracts. The highest levels of gallic acid were found in the Hainan 2011 cocoa beans followed by the samples from Papua New Guinea. The differences in values may be the result of the fermentation process and the storage period. Each stage in the processing of cocoa alters the chemistry of polyphenols. During cocoa fermentation, polyphenols are subjected to biochemical modifications through oxidation and polymerization and to binding with proteins, thereby decreasing their solubility and astringency effects [19]. Subsequently, during drying, the amount of polyphenols is substantially reduced mainly by enzymatic browning [14]. On the contrary, the roasting process, which is responsible for reducing bitter and acidic tastes, causes small changes in polyphenolic concentration. Several studies showed a correlation between antioxidant activity and phenolic content [23]. 

### 3.5. Total and Free Amino Acids

Aroma precursors in cocoa beans, which include free amino acids, peptides and reducing sugars, develop into cocoa-specific aroma through Maillard reactions during roasting. The concentrations of total, hydrophobic, acidic and other free amino acids in dried cocoa beans are presented in Table 3, Table 4. In the study, the average total amino acid content ranged from 11.58 g/100 g to 18.17 g/100 g. The total amino acid content was the highest in the Indonesian unfermented sample, followed by the Hainan 2011 and Papua New Guinea samples. The levels found in the Hainan 2010 sample was higher than those found in the Indonesian fermented sample, which had the lowest total amino acid content.

**Table 3:** The concentration of total amino acids (g/100 g).

Amino acids	C1 (g/100 g)	C2 (g/100 g)	C3 (g/100 g)	C4 (g/100 g)	C5 (g/100 g)
asp	1.48234112	1.248726	2.03329	1.638016	2.285384
glu	2.76421738	2.33606	3.61903	2.745753	3.024123
ser	0.66305973	0.567914	0.928503	0.797482	0.934248
his	0.27536076	0.231274	0.392731	0.290148	0.342329
gly	0.67713588	0.553377	0.85647	0.71582	0.873638
thr	0.58388107	0.515058	0.778265	0.684044	0.812028
arg	0.96300469	0.884542	1.344468	1.123294	1.302416
ala	0.72384247	0.549737	0.911206	0.675514	0.82077
tyr	0.40220555	0.376532	0.569141	0.481996	0.507722
cys-s	0.09059641	0.103466	0.134149	0.14175	0.144297
val	0.88030156	0.772049	1.147787	0.929087	1.15285
met	0.16747286	0.069999	0.204375	0.1672	0.213946
phe	0.75009401	0.659956	1.148313	0.837903	0.958442
ile	0.56001189	0.503279	0.714016	0.610186	0.740686
leu	0.91887279	0.80002	1.254947	1.036056	1.242061
lys	0.6899189	0.685431	1.130527	0.802536	1.032648
pro	0.85167766	0.723573	1.004048	0.837604	0.858958
Total	13.4439947	11.58099	18.17126	14.51439	17.24655

**Table 4:** The concentration of free amino acids (g/100 g).

Amino acids	C1 (g/100 g)	C2 (g/100 g)	C3 (g/100 g)	C4 (g/100 g)	C5 (g/100 g)
asp	0.06175624	0.052118	0.0595	0.03942	0.080142
glu	0.0616727	0.061372	0.076922	0.034869	0.064857
ser	0.00145676	0.002074	0.000417	0.000607	0.001391
his	0.00334901	0.003545	0.006681	0.002198	0.012053
gly	0.00981977	0.007367	0.017332	0.006294	0.023166
thr	0.01156708	0.011032	0.013705	0.008447	0.022726
arg	0.03857273	0.061132	0.017908	0.025347	0.095447
ala	0.11614724	0.073076	0.079882	0.047004	0.112538
tyr	0.04184931	0.0555	0.034151	0.029755	0.077396
cys-s	0.00110326	0.001001	1.25E-07	0.001071	0.001652
val	0.07068854	0.064691	0.054895	0.052798	0.11922
met	0.0015651	0.002192	0.000395	0.000734	0.003633
phe	0.06475303	0.083083	0.020958	0.050539	0.146415
ile	0.03503073	0.034275	0.019892	0.029651	0.073133
leu	0.09972465	0.117406	0.036901	0.061232	0.168642
lys	0.02884247	0.04466	0.018216	0.017223	0.051872
pro	0.05347026	0.043259	0.055476	0.048206	0.102607
Total	0.70136889	0.717783	0.513232	0.455394	1.15689

During cocoa fermentation, proteolysis, catalyzed by aspartic endoprotease and carboxypeptidase, gives rise to amino acids and oligopeptides. The aspartic endoprotease from cocoa beans cleaves protein substrate preferentially at hydrophobic amino acid residues to produce oligopeptides with hydrophobic amino acid residues at their carboxy terminal ends [10].

The predominant release of hydrophobic free amino acids during proteolysis results in the high proportion of these amino acids after fermentation. The concentration of hydrophobic free amino acids generally increased in the samples added with carboxypeptidase as incubation progressed, whereas in samples without carboxypeptidase, the concentration of hydrophobic free amino acids was relatively constant. Kirchhoff *et al.* [24] found that hydrophobic free amino acids, such as leucine, phenylalanine, alanine and tyrosine, contribute to the formation of cocoa flavor. These free amino acids are presumably the result of the specific activity of aspartic endoprotease, which splits proteins into hydrophobic peptides, allowing a limited action of carboxypeptidase to release predominantly hydrophobic free amino acids.

### 3.6. Flavor with Electronic Nose (E-nose)

**Figure 3:**
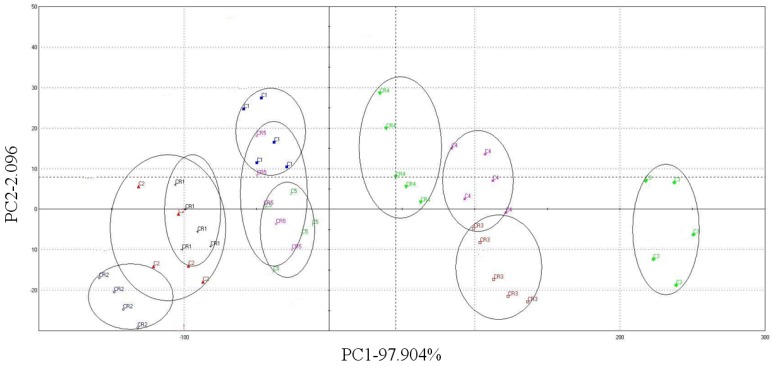
Principal component analysis of the data presented for PC1 and PC2.

The e-nose clearly distinguished all the different cocoa bean samples using PCA as a data treatment technique (Figure 3). PCA, associated with linear correlation, was applied to the complete collection of sensor signals. The set of maximum signal values for each sensor resulted in a six-dimensional (six sensors) pattern or data vector. The PCA plot showed the associations of large percentage variances in PC scores of 1 and 2 are 97.904% and 2.096%, respectively. These values represented variability in major constituents. The advantages of proper data treatment techniques include greater flexibility, smaller processing times, enhanced performances and better interpretation of the results [25].

Examining a two-dimensional score plot in the space defined as PC1 and PC2, one can deduce that the distribution of samples followed a pattern. As each cluster clearly separated, unfermented samples could be distinguished from fermented samples, but a clear differentiation between all ten samples was not possible. Remarkably, the values of most fermented samples were placed in the negative part of the *x*-axis, whereas the values for the unfermented Indonesian cocoa beans and the Hainan 2010 cocoa beans were placed in the positive part. On the *y*-axis, no clear differentiation was possible. The separation between groups is related to the origin and processing of beans. The right group comprised four cocoa samples that represented mostly unfermented cocoa beans and possessed less chocolate flavor. The left group (six samples) represented mostly fermented and roasted cocoa beans, but the e-nose could not discriminate the fermented and roasted cocoa beans from Indonesia and Papua New Guinea. The phenomenon may be caused by the flavor of volatile acid. During cocoa bean fermentation, the role of micro-organisms is limited to the removal of the pulp that surrounds the fresh beans and to the production of indispensable metabolites [26,27]. The former involves pectin depolymerization by yeasts. The latter encompasses anaerobic yeast fermentation of sugars to ethanol, microaerophilic fermentation of sugars and citric acid to lactic acid, acetic acid and mannitol by lactic acid bacteria and aerobic exothermic bioconversion of ethanol into acetic acid by acetic acid bacteria [28,29,30].

## 4. Conclusions

The varieties, growing conditions and fermentation of cocoa beans seem to influence their average bean weight, level of polyphenols and amino acids. Such observation held true, as the present study found varying values in the cocoa bean samples used. Although the Hainan cocoa beans were lighter than the other samples, their total polyphenolic and flavonoid content were higher or equal to the cocoa beans from Papua New Guinea and Indonesia. Aside from the interest in the quality standards and economic specification of raw material, manufacturers today pay great attention to declaring their products as functional food. Thus, our data provide additional knowledge to be considered in the promotion of cocoa plantations in China. 

The results obtained from this study are essential in understanding and solving the problems associated with the quality of raw cocoa beans. Further research is needed to determine the effect of the growing conditions, storage time and fermentation on the physico-chemical and flavor quality attributes of industrial raw cocoa material. Such a direction is intended for improving the quality of raw cocoa beans sourced from China, Indonesia and Papua New Guinea.
